# Construction and validation of a risk prediction model for hyperamylasemia after kidney transplantation

**DOI:** 10.3389/fimmu.2025.1675844

**Published:** 2025-10-30

**Authors:** Linde Li, Guifeng Dang, Feiyi Du, Meisi Li, Qianhua Ma, Ning Wen, Jiqiu Wen, Jianhui Dong, Xuyong Sun

**Affiliations:** Institute of Transplant Medicine, The Second Affiliated Hospital of Guangxi Medical University, Guangxi Clinical Research Center for Organ Transplantation, Guangxi Key Laboratory of Organ Donation and Transplantation, Nanning, China

**Keywords:** kidney transplantation, hyperamylasemia, nomogram, internal validation, prediction

## Abstract

**Background:**

Kidney transplantation (KT) is the preferred treatment for patients with end-stage renal disease (ESRD); however, postoperative hyperamylasemia (HA) remains common and has been associated with acute rejection (AR), infection, and impaired graft function. Early identification of HA risk factors is essential to improve outcomes of kidney transplant recipients (KTR). This study aimed to develop and internally validate a novel nomogram for predicting the risk of HA after KT, thereby supporting personalized monitoring, prevention and intervention strategies.

**Methods:**

We retrospectively analyzed KTR treated at the Transplant Medicine Institution of the Second Affiliated Hospital of Guangxi Medical University from July 2021 to June 2022. Based on admission dates, patients were assigned to a training cohort (n=243, July 2021 to March 2022) and a validation cohort (n=107, April 2022 to June 2022). In the training cohort, risk factors of HA were identified using logistic regression, Lasso regression and clinical consideration. Subsequently, a nomogram was developed to predict HA risk in patients who underwent KT based on the identified variables. Model performance was evaluated using receiver operating characteristic (ROC) curves, calibration plots, and decision curve analysis (DCA).

**Results:**

A total of 350 KTR and their corresponding 182 donors were enrolled in this study. The nomogram incorporated six predictive factors: recipient preoperative white blood cell (WBC) count, induction, tacrolimus (FK506) trough concentration, AR, donor age, and donor total bilirubin (TBIL) level according to results of logistic regression, Lasso regression and clinical consideration. The nomogram showed moderate predictive performance, with an area under the ROC curve (AUC) of 0.730 (Youden index = 0.683) in the training cohort and 0.731 (Youden index = 0.767) in the validation cohort. Furthermore, calibration plots indicated close agreement between predicted and actual outcomes, and DCA confirmed net clinical benefit across a range of threshold probabilities.

**Conclusions:**

A novel nomogram was established to predict HA after KT, which may support early risk stratification and personalized management of KTR. External multicenter validation is needed before clinical implementation.

## 1 Introduction

KT stands as the optimal therapeutic intervention for patients with ESRD, delivering superior patients’ survival and graft longevity than maintenance dialysis (1). However, even with ongoing advances in surgical techniques and immunosuppressive regimens, KTR remain susceptible to numerous postoperative complications. These risks underline the necessity for robust, continuous laboratory surveillance to enable early detection and management of adverse events (2). Among routinely monitoring biomarkers, serum amylase has historically been considered an indicator of pancreatic injury. Notably, elevated serum amylase, HA, is remarkably common, frequently emerging in the absence of clinical pancreatitis or abdominal symptoms (3). This phenomenon challenges conventional diagnostic thresholds and underscores the complexity of interpreting biochemical abnormalities in KTR.

The pathophysiology of HA after KT is multifaceted. Amylase, a hydrolase involved in carbohydrate digestion, includes pancreatic (P-type) and salivary (S-type) isoenzymes, both primarily eliminated through renal filtration (4, 5). The decline in native kidney function both pre- and post-transplantation markedly reduces amylase clearance, which may result in persistently elevated serum levels unrelated to pancreatic disease (6, 7). Recent studies further suggest that minor elimination pathways, such as hepatic and reticuloendothelial systems, influence amylase kinetics in patients with systemic comorbidities, particularly following organ transplantation.

HA is reported in 20%-61% of KTR, varying by diagnostic criteria and timing of assessment (6, 8, 9). Most cases are transient and asymptomatic, but a proportion may signal critical complications, such as AR, infection, acute pancreatitis, or impending graft dysfunction. Notably, the true prognostic significance of isolated HA in this setting remains undefined. The absence of standardized diagnostic criteria contributes to clinical uncertainty, often leading to both under-recognition of real threats and unnecessary alarm when isolated biochemical changes are found.

Despite these challenges, risk stratification for post-transplant HA remains infrequently standardized or data-driven. Due to variations in laboratory testing methods, differences in patient populations, and the impact of renal insufficiency on serum amylase levels, the definition of HA ranges from 100U/L to 132U/L across studies (10–12). Previous studies have been constrained by small sample sizes, heterogeneous definitions, and methodological limitations, which have impeded their translation into widely applicable clinical guidelines. In this context, the clinical interpretation of HA is inherently complex. Existing evidence suggests that the etiology of post-transplant HA is multifactorial, potentially involving factors such as impaired renal clearance of amylase, perioperative stress, immunosuppressive therapy, and subclinical pancreatic injury (13–15). However, the relative contributions and interactions of these factors remain poorly understood. Notably, there is a lack of large-scale, well-designed studies specifically examining the determinants of HA in this patient population.

There is a pressing need for a validated, individualized risk prediction tool that integrates diverse recipient and donor variables—including inflammatory status, immunosuppressive regimens, and donor organ quality—to guide early detection, tailored monitoring, and targeted intervention for HA in KTR.

In this context, our study developed and internally validated a novel nomogram to quantify HA risk in the early postoperative period post-KT. By applying contemporary statistical modeling to a well-characterized cohort, we aimed to deliver a practical instrument that not only advanced the precision of risk assessment but also supported individualized patient management. This model may serve as a foundation for future external validation and for the evolution of personalized perioperative strategies in KT.

## 2 Materials and methods

### 2.1 Study design

This study was conducted with a cohort of 403 KTR at the Transplant Medical Institution of the Second Affiliated Hospital of Guangxi Medical University from July 2021 to June 2022. After screening, we excluded patients based on the following criteria: preoperative HA, history of pancreatitis, living donor KT, combined organ transplant, and incomplete clinical data. As a result, 350 KTR and their corresponding 182 donors were included in the final analysis.

The 350 KTR were divided into two cohorts based on the date of admission: a training cohort (n=243; July 2021 to March 2022) and a validation cohort (n=107; April 2022 to June 2022).

### 2.2 Patient selection

The inclusion criteria for KTR were as follows: (1) diagnosis of chronic kidney disease stage 5 (CKD-5); (2) first-time KT; (3) age between 18 and 70 years; and (4) stable preoperative cardiopulmonary function sufficient to tolerate surgery.

The exclusion criteria for recipients were as follows: (1) recipients of multi-organ combined transplantation; (2) patients with a preoperative history of pancreatitis or HA; and (3) patients with more than 20% missing clinical data.

The inclusion criteria for donors included: (1) deceased citizen donors with verified identity; (2) organ donation conducted only after the donor’s family members or authorized representatives signed both the China Human Organ Donation Registration Form and the Informed Consent Form for Human Organ Donation; (3) donors who met established brain death criteria; and (4) donors with complete clinical data available.

The exclusion criteria for donors were: (1) living donors; (2) donors with a history of acute pancreatitis prior to donation.

### 2.3 Data collection

Recipient data included: gender, age, body mass index (BMI), dialysis method (hemodialysis [HD] or peritoneal dialysis [PD]), hospitalization duration, dialysis duration, history of hypertension, hyperparathyroidism (HPT), surgical history, transfusion history, smoking history, alcohol consumption history, operation time, blood type, preoperative blood routine tests, electrolytes, procalcitonin (PCT), C-reactive protein (CRP), human leukocyte antigen (HLA) antibodies, blood glucose, blood lipids, perioperative immunosuppression protocol, induction immunosuppressive medications, Whether to add hormone pulse therapy, occurrence of delayed graft function (DGF), occurrence of AR, and FK506 serum drug concentration. Among these, AR was defined as cases diagnosed by clinical symptoms, laboratory indicators, and/or pathological examination before the confirmation of HA (The diagnosis of AR was made with specific reference to the KDIGO Clinical Practice Guideline on the Care of Kidney Transplant Recipients). In this study, the serum trough concentration of FK506 specifically referred to the latest trough concentration measured during the routine postoperative monitoring period before the confirmation of HA.

Donor data included: gender, age, BMI, cause of death, Intensive Care Unit (ICU) treatment duration, Organ Procurement Organization (OPO) intervention time, cold ischemia time (CIT), history of cardio-pulmonary resuscitation (CPR) prior to procurement, infectious disease screening, etiological examination, complete blood count before procurement, CRP, PCT, TBIL, Direct Bilirubin (DBIL), Indirect Bilirubin (IBIL), Alanine Aminotransferase (ALT), Aspartate Aminotransferase (AST), blood glucose, renal function, and electrolytes.

### 2.4 Immunosuppressive regimen

The development of the immune induction regimen was grounded in a comprehensive preoperative evaluation of the recipient’s immunological risk. Rabbit anti-human thymocyte globulin (rATG) was administered as intraoperative induction for recipients with high-risk factors such as pre-existing Donor-Specific Antibody (DSA) and Panel Reactive Antibody (PRA) levels >50%. Basiliximab (BAS) was used as intraoperative induction for recipients with immunological low risk. Anti-Thymocyte Globulin Porcine (ATG) was intraoperatively utilized for recipients with middle risk. FK506 combined with mycophenolate and corticosteroids remains the most commonly used maintenance regimen in our institution.

### 2.5 Diagnosis of HA after KT

Currently, no standardized criteria exist for defining post-kidney transplantation hyperamylasemia (HA). In this study, HA was defined as serum amylase levels >110 U/L in two tests within 6–24 hours after surgery, without clinical symptoms such as abdominal pain. This time window was chosen to capture early postoperative enzyme changes while reducing transient, stress-related fluctuations within the first 6 hours. The 110 U/L threshold was based on our laboratory’s reference range and adjusted for renal impairment in transplant recipients that may affect amylase clearance. This definition balances sensitivity and specificity while accounting for methodological and physiological variability in this population (16).

### 2.6 Nomogram construction and validation

#### 2.6.1 Dataset partitioning and data processing

The dataset was divided into a training cohort (July 2021 to March 2022, N = 243) and a validation cohort (April 2022 to June 2022, N = 107) based on admission dates. Baseline characteristics were analyzed for the overall population, training cohort, and validation cohort using Stata 16.0 (Stata Corp, College Station, TX, USA). Categorical variables were compared using the chi-square test. For continuous variables, normality was assessed prior to analysis. Normally distributed variables with homogeneous variances were analyzed using the independent samples t-test (mean [standard deviation]); if variances were unequal, Welch’s t-test was applied. Non-normally distributed continuous variables were analyzed using the Wilcoxon rank-sum test (median [interquartile range]). The comparability between the two cohorts was assessed to confirm model applicability. All missing data were confirmed to be missing completely at random (MCAR). Variables with more than 20% missingness (e.g., mycophenolic acid concentration, cytokine levels, G-test results) were excluded to minimize bias. For variables with less than 20% missingness, multiple imputation using chained equations (MICE) was performed to preserve multivariate distribution and inter-variable correlations. This preprocessing strategy enhanced data completeness and ensured the robustness and validity of the statistical inferences.

#### 2.6.2 Variable screening and model establishment

Univariate logistic regression analysis was conducted using SPSS 26.0 to identify potential risk factors for post-transplant HA in the training cohort, with variables having a P-value < 0.1 considered for inclusion. Subsequently, Lasso regression was applied using R 4.2.1 to perform variable selection and dimensionality reduction. The optimal penalty parameter (λ) was determined through 10-fold cross-validation. This was followed by stepwise multivariate logistic regression to build the final predictive model, using an inclusion criterion of α = 0.05 and an exclusion criterion of α = 0.1.

#### 2.6.3 Model validation

Internal validation was conducted using the predefined validation cohort. Model performance was comprehensively assessed in terms of discrimination, calibration, and clinical utility. Discrimination was evaluated by calculating the area under the receiver operating characteristic curve (AUC). Calibration was assessed using the Hosmer–Lemeshow goodness-of-fit test. Clinical utility was evaluated using DCA to estimate the net benefit across a range of threshold probabilities.

All statistical analyses were conducted using Stata 16.0, SPSS 26.0, R version 4.2.1, and RStudio. A two-sided P-value < 0.05 was considered statistically significant.

## 3 Results

### 3.1 Participant flow and baseline characteristics

**Figure 1:**
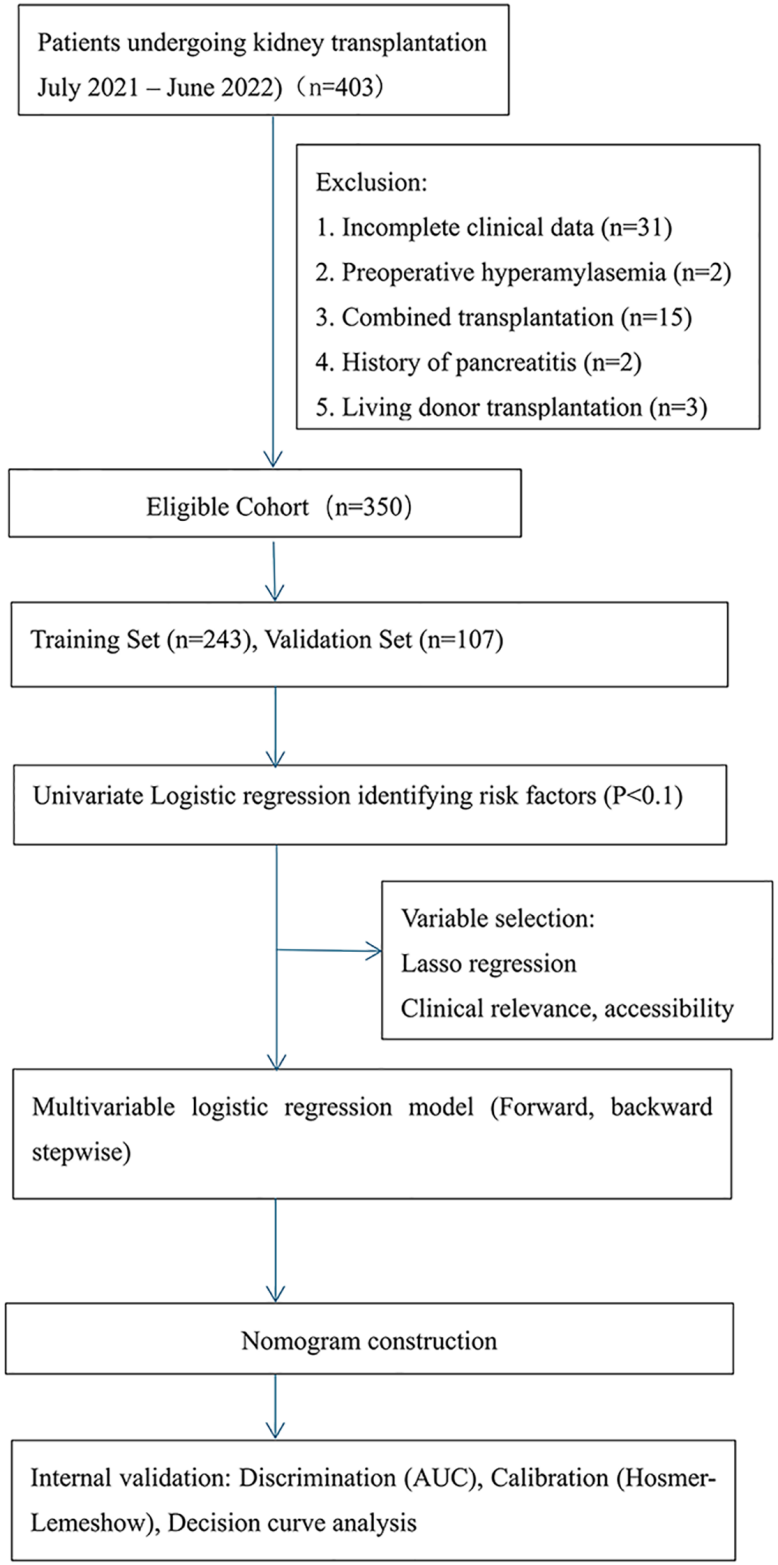
Study flowchart illustrating patient selection and predictive model construction for KTR.

Of 403 screened KTR, 350 met the inclusion criteria and were analyzed (**Figure 1**). The training cohort included 243 patients and the validation cohort 107. Baseline characteristics are shown in **Table 1**. Among these, 181 (51.7%) developed HA. Comparative analysis between the HA group and the normal amylase group revealed several noteworthy clinical differences. Notably, patients in the HA group had a significantly longer median hospital stay (10 days vs. 9 days, p < 0.01), indicating a more complex or prolonged postoperative recovery. Additionally, lower incidence of acute rejection (AR) was found in the HA group (2.2% vs. 7.1%, p = 0.04); FK506 trough concentrations were significantly higher in the HA group (median 7.1 ng/mL vs. 6.3 ng/mL, p < 0.01). These differences suggested that adequate FK506 levels is critical in reducing the risk of AR, also contributing to enzymatic alterations.

**Table 1:** Baseline characteristics of study participants.

Variable	Total (N = 350)	Normal amylase group (N = 169)	Hyperamylasemia group (N = 181)	*P*-value
Recipient				
Gender				0.31
Female	115	51 (30.2%)	64 (35.4%)	
Male	235	118 (69.8%)	117 (64.6%)	
Age (year)	39.0 (32.0,49.0)	38.0 (31.0, 47.0)	40.0 (32.0, 49.0)	0.30
BMI (kg/m^2^)	22.1 (19.7,24.5)	22.2 (20.2, 24.5)	22.0 (19.5, 24.5)	0.65
Dialysis modality				<0.01
Not dialyzed	5	1 (0.6%)	4 (2.2%)	
HD	269	131 (77.5%)	138 (76.2%)	
PD	41	27 (16.0%)	14 (7.7%)	
HD+PD	35	10 (5.9%)	25 (13.8%)	
Hospital stay duration (day)	10 (8.0,15.0)	9.0 (7.0, 12.0)	10.0 (8.0, 16.0)	<0.01
Dialysis duration (month)	24 (14.0,48.0)	24.0 (15.0, 41.0)	26.0 (14.0, 48.0)	0.74
Hypertension				0.27
No	47	19 (11.2%)	28 (15.5%)	
Yes	303	150 (88.8%)	153 (84.5%)	
HPT				0.02
No	185	100 (50.2%)	85 (47%)	
Yes	165	69 (40.8%)	96 (53.0%)	
Surgical history				0.41
No	246	115 (68.0%)	131 (72.4%)	
Yes	94	54 (32.0%)	50 (27.6%)	
History of blood transfusion				0.70
No	273	130 (76.9%)	143 (79.0%)	
Yes	77	39 (23.1%)	38 (21.0%)	
Smoking history				0.27
No	286	134 (79.3%)	152 (84.0%)	
Yes	64	35 (20.7%)	29 (16.0%)	
Drinking history				0.32
No	291	137 (81.1%)	154 (85.1%)	
Yes	59	32 (18.9%)	27 (14.9%)	
HLA antibodies				1.00
NO	323	156 (92.3%)	167 (92.3%)	
Yes	27	13 (7.7%)	14 (7.7%)	
Operation time (min)	165 (144,185)	163.0 (146.0, 185.0)	165.0 (140.0, 185.0)	0.51
Blood type				0.17
A	76	41 (24.3%)	35 (19.3%)	
B	73	27 (16.0%)	46 (25.4%)	
O	173	87 (51.5%)	86 (47.5%)	
AB	28	14 (8.3%)	14 (7.7%)	
HLA antibodies				1.00
NO	323	156 (92.3%)	167 (92.3%)	
Yes	27	13 (7.7%)	14 (7.7%)	
Blood glucose				0.38
Normal	295	139 (82.2%)	156 (86.2%)	
Abnormal	55	30 (17.8%)	25 (13.8%)	
Blood lipid				0.34
Normal	164	84 (49.7%)	80 (44.2%)	
Abnormal	186	85 (50.3%)	101 (55.8%)	
Maintenance regimen				0.31
MPA+FK506 +Pred	310	153 (90.5%)	157 (86.7%)	
MMF+FK506 +Pred	40	16 (9.5%)	24 (13.3%)	
Induction				0.06
BAS	238	110 (65.1%)	128 (70.7%)	
ATG	95	54 (32.0%)	41 (22.7%)	
rATG	17	5 (3.0%)	12 (6.6%)	
Hormone pulse				0.88
No	303	147 (87.0%)	156 (86.2%)	
Yes	47	22 (13.0%)	25 (13.8%)	
DGF				0.70
No	320	156 (92.3%)	164 (90.6%)	
Yes	30	13 (7.7%)	17 (9.4%)	
AR				0.04
No	334	157 (92.9%)	177 (97.8%)	
Yes	16	12 (7.1%)	4 (2.2%)	
FK506 trough concentration (ng/ml)	6.6 (4.8, 8.9)	6.3 (4.5, 8.4)	7.1 (5.1, 9.4)	<0.01
Donor				
Age (year)	48 (37,55)	46.0 (35.0, 54.0)	48.0 (38.0, 56.0)	0.03
Infectious markers				0.73
Normal	313	150 (88.8%)	163 (90.1%)	
Abnormal	37	19 (11.2%)	18 (9.9%)	
Pathogens				0.47
Negative	294	139 (82.2%)	155 (85.6%)	
Positive	56	30 (17.8%)	26 (14.4%)	
Gender				0.51
Female	74	33 (19.5%)	41 (22.7%)	
Male	276	136 (80.5%)	140 (77.3%)	
BMI (kg/m^2^)	23.5 (22,25)	23.5 (21.3, 25.3)	23.5 (22.0, 25.1)	0.39
ICU length of stay (day)	5.6 (3.3,10)	5.8 (3.3, 10.8)	5.5 (3.2, 9.2)	0.43
OPO intervention time (hour)	37.9 (24,55)	36.0 (21.1, 45.2)	38.5 (28.3, 56.0)	0.08
Cold ischemia time (hour)	10 (8.5, 12.3)	10.0 (8.6, 12.4)	10.0 (8.5, 12.3)	0.99
History of CPR				0.89
No	291	140 (82.8%)	151 (83.4%)	
Yes	59	29 (17.2%)	30 (16.6%)	

Furthermore, the prevalence of hyperparathyroidism was significantly higher in the HA group (53.0% vs. 40.8%, p = 0.02), suggesting a potential association between HA and post-transplant metabolic disturbances. In contrast, no statistically significant differences were observed between the HA and normal amylase groups regarding the incidence of DGF. These findings suggest that HA may could reflect underlying perioperative metabolic disturbances or subclinical pancreatic inflammation.

Taken together, these results underscore the multifactorial etiology of HA, which likely involves interactions between the recipient’s inflammatory status, immunosuppressive therapy, and donor-related factors. The findings emphasize the clinical relevance of incorporating HA into perioperative risk stratification and management strategies for KTR.

### 3.2 Baseline characteristics of training and validation cohort

**Table 2** compares baseline characteristics between normal amylase and HA groups within the training cohort (N = 243) and validation cohort (N = 107). Most demographic and clinical variables, including age, gender, BMI, blood type, dialysis duration, hypertension, surgical history, transfusion history, and donor factors, were well balanced between groups in both cohorts.

**Table 2:** Baseline characteristics of training and validation cohorts.

Variable	Training cohort (n=243)			Validation cohort (n=107)		
	Normal amylase group (n=126)	Hyperamylasemia group (n=117)	*P*-value	Normal amylase group(n=43)	Hyperamylasemia group (n=64)	*P* -value
Preoperative recipient						
Gender			0.13			0.54
Female	33 (26.2%)	42 (35.9%)		18 (41.9%)	22 (34.4%)	
Male	93 (73.8%)	75 (64.1%)		25 (58.1%)	42 (65.6%)	
Age (year)			0.05			0.80
≤18	6 (4.8%)	2 (1.7%)		0 (0.0%)	1 (1.6%)	
19-44	82 (65.1%)	65 (55.6%)		30 (69.8%)	40 (62.5%)	
45-60	33 (26.2%)	48 (41.0%)		11 (25.6%)	21 (32.8%)	
≥ 61	5 (4.0%)	2 (1.7%)		2 (4.6%)	2 (3.1%)	
BMI (kg/m^2^)			0.26			0.78
<18.49	13 (10.3%)	20 (17.1%)		6 (14.0%)	7 (10.9%)	
18.50-24.99	86 (68.3%)	70 (59.8%)		29 (67.4%)	44 (68.8%)	
25.00-29.99	24 (19.0%)	21 (17.9%)		8 (18.6%)	11 (17.2%)	
>30.00	3 (2.4%)	6 (5.1%)		0 (0.0%)	2 (3.1%)	
Dialysis modality			0.12			0.03
No dialysis	1 (0.8%)	2 (1.7%)		0 (0.0%)	2 (3.1%)	
HD	98 (77.8%)	92 (78.6%)		33 (76.7%)	46 (72.0%)	
PD	18 (14.3%)	8 (6.8%)		9 (20.9%)	6 (9.3%)	
HD+PD	9 (7.1%)	15 (12.8%)		1 (2.4%)	10 (15.6%)	
Blood type			0.35			0.11
A	28 (22.2%)	23 (19.7%)		13 (30.2%)	12 (18.8%)	
B	21 (16.7%)	28 (23.9%)		6 (14.0%)	18 (28.1%)	
O	65 (51.6%)	60 (51.3%)		22 (51.2%)	26 (40.6%)	
AB	12 (9.5%)	6 (5.1%)		2 (4.6%)	8 (12.5%)	
HLA antibodies			1.00			1.00
No	118 (93.7%)	110 (94.0%)		38 (88.4%)	57 (89.1%)	
Yes	8 (6.3%)	7 (6.0%)		5 (11.6%)	7 (10.9%)	
WBC (×10^9^/L)	6.2 (4.8, 8.1)	7.4 (6.0, 8.8)	<0.01	6.5 (5.1, 8.0)	6.1 (5.2, 7.8)	0.57
NEU (%)	0.7 (0.6, 0.8)	0.7 (0.6, 0.8)	0.24	0.7 (0.6, 0.8)	0.7 (0.6, 0.8)	0.49
Ca^2+^(mmol/L)	2.3 (2.2, 2.4)	2.3 (2.2, 2.5)	0.38	2.3 (2.1, 2.4)	2.2 (2.0, 2.4)	0.74
PCT (ng/ml)	0.5 (0.3, 1.1)	0.6 (0.3, 1.4)	0.40	0.3 (0.2, 0.8)	0.4 (0.2, 0.6)	0.54
CRP (mg/L)	4.8(2.2, 10.1)	3.8 (1.8, 8.0)	0.20	2.5 (1.5, 5.4)	2.9 (1.6, 4.9)	0.60
Dialysis duration (month)			0.25			0.56
≤34	68 (54.0%)	54 (46.2%)		20 (46.5%)	34 (53.1%)	
≥35	58 (46.0%)	63 (53.8%)		23 (53.5%)	30 (46.9%)	
Blood glucose			0.51			0.75
Normal	101 (80.2%)	98 (83.8%)		38 (88.4%)	58 (90.6%)	
Abnormal	25 (19.8%)	19 (16.2%)		5 (11.6%)	6 (9.4%)	
Blood lipid			0.37			0.84
Normal	63 (50.0%)	51 (43.6%)		21 (48.8%)	29 (45.3%)	
Abnormal	63 (50.0%)	66 (56.4%)		22 (51.2%)	35 (54.7%)	
Hypertension			0.47			0.35
No	16 (12.7%)	19 (16.2%)		3 (7.0%)	9 (14.1%)	
Yes	110 (87.3%)	98 (83.8%)		40 (93.0%)	55 (85.9%)	
HPT			0.03			0.82
No	90 (71.4%)	68 (58.1%)		10 (23.3%)	17 (26.6%)	
Yes	36 (28.6%)	49 (41.9%)		33 (76.7%)	47 (73.4%)	
Surgical history			0.12			0.68
No	85 (67.5%)	90 (76.9%)		30 (69.8%)	41 (64.1%)	
Yes	41 (32.5%)	27 (23.1%)		13 (30.2%)	23 (35.9%)	
History of blood transfusion			0.36			0.47
No	94 (74.6%)	94 (80.3%)		36 (83.7%)	49 (76.6%)	
Yes	32 (25.4%)	23 (19.7%)		7 (16.3%)	15 (23.4%)	
Smoking history			0.41			0.61
No	100 (79.4%)	98 (83.8%)		34 (79.1%)	54 (84.4%)	
Yes	26 (20.6%)	19 (16.2%)		9 (20.1%)	10 (15.6%)	
Drinking history			0.23			1.00
No	101 (80.2%)	101 (86.3%)		36 (83.7%)	53 (82.8%)	
Yes	25 (19.8%)	16 (13.7%)		7 (16.3%)	11 (17.2%)	
Perioperative Recipients						
DGF			0.44			1.00
No	113 (89.7%)	101 (86.3%)		43 (100.0%)	63 (98.4%)	
Yes	13 (10.3%)	16 (13.7%)		0 (0.0%)	1 (1.6%)	
AR			0.07			1.00
No	114 (90.5%)	113 (96.6%)		43 (100.0%)	64 (100.0%)	
Yes	12 (9.5%)	4 (3.4%)		0 (0.0%)	0 (0.0%)	
Operation time (min)			0.70			0.69
≤172	69 (54.8%)	61 (52.1%)		18 (41.9%)	30 (46.9%)	
>172	57 (45.2%)	56 (47.9%)		25 (58.1%)	34 (53.1%)	
Hospital stay duration(day)			0.11			0.32
≤13	83 (65.9%)	65 (55.6%)		25 (58.1%)	30 (46.9%)	
≥ 14	43 (34.1%)	52 (44.4%)		18 (41.9%)	34 (53.1%)	
Maintenance regimen			0.24			0.82
MPA+FK506 +Pred	122 (96.8%)	109 (93.2%)		31 (72.1%)	48 (75.0%)	
MMF+FK506 +Pred	4 (3.2%)	8 (6.8%)		12 (27.9%)	16 (25.0%)	
Induction			0.02			0.65
BAS	82 (65.1%)	81 (69.2%)		28 (65.1%)	47 (73.4%)	
ATG	42 (33.3%)	27 (23.1%)		12 (27.9%)	14 (21.9%)	
rATG	2 (1.6%)	9 (7.7%)		3 (7.0%)	3 (4.7%)	
Hormone pulse			0.58			0.76
No	110 (87.3%)	99 (84.6%)		37 (86.1%)	57 (89.1%)	
Yes	16 (12.7%)	18 (15.4%)		6 (13.9%)	7 (10.9%)	
FK506 trough concentration (ng/ml)	6.4 (4.6, 8.6)	7.0 (4.9, 9.1)	0.12	6.2 (4.2, 8.0)	7.3 (5.4, 10.5)	0.01
CRP (mg/L)	25.9(16.7,41.2)	26.9 (19.2, 43.7)	0.32	21.9(14.1,38.1)	23.5 (17.2, 35.7)	0.79
PCT (ng/mL)	1.5 (0.6, 3.6)	2.6 (0.8, 7.1)	<0.01	2.1 (0.6, 3.5)	2.1 (0.6, 4.1)	0.81
WBC (×10^9^/L)	13.8(11.2,16.8)	15.5 (13.4, 18.3)	<0.01	15.0(13.2,20.2)	14.9 (11.9,18.9)	0.65
NEU (%)	0.9 (0.9, 1.0)	0.9 (0.9, 1.0)	0.04	1.0 (0.9, 1.0)	0.9 (0.9, 1.0)	0.09
Donor						
Age (year)			0.10			0.61
≤18	15 (11.9%)	5 (4.3%)		2 (4.7%)	4 (6.2%)	
19-44	41 (32.5%)	35 (29.9%)		19 (44.2%)	20 (31.3%)	
45-60	61 (48.4%)	63 (53.8%)		19 (44.2%)	33 (51.6%)	
≥ 61	9 (7.1%)	14 (12.0%)		3 (6.9%)	7 (10.9%)	
Infectious markers			0.14			0.23
Normal	110 (87.3%)	109 (93.2%)		40 (93.0%)	54 (84.4%)	
Abnormal	16 (12.7%)	8 (6.8%)		3 (7.0%)	10 (15.6%)	
Cold ischemia time (hour)			0.90			0.44
<10.58 h	63 (50.0%)	57 (48.7%)		20 (46.5%)	35 (54.7%)	
≥10.58h	63 (50.0%)	60 (51.3%)		23 (53.5%)	29 (45.3%)	
Pathogens			0.21			0.80
Negative	103 (81.7%)	103 (88.0%)		36 (83.7%)	52 (81.3%)	
Positive	23 (18.3%)	14 (12.0%)		7 (16.3%)	12 (18.7%)	
Gender			1.00			0.36
Female	25 (19.8%)	24 (20.5%)		8 (18.6%)	17 (26.6%)	
Male	101 (80.2%)	93 (79.5%)		35 (81.4%)	47 (73.4%)	
BMI (kg/m^2^)			0.73			0.86
<18.49	6 (4.8%)	7 (6.0%)		2 (4.7%)	4 (6.2%)	
18.50-24.99	86 (68.3%)	81 (69.2%)		32 (74.4%)	43 (67.2%)	
25-29.99	31 (24.6%)	24 (20.5%)		8 (18.6%)	14 (21.9%)	
>30	3 (2.4%)	5 (4.3%)		1 (2.3%)	3 (4.7%)	
ICU length of stay (day)			0.52			1.00
≤5.6	60 (47.6%)	61 (52.1%)		21 (48.8%)	32 (50.0%)	
>5.61	66 (52.4%)	56 (47.9%)		22 (51.2%)	32 (50.0%)	
OPO intervention time (hour)			0.09			0.54
≤37.85	77 (61.1%)	58 (49.6%)		18 (41.9%)	22 (34.4%)	
>37.85	49 (38.9%)	59 (50.4%)		25 (58.1%)	42 (65.6%)	
WBC (×10^9^/L)	14.4(10.7,18.6)	12.6(8.3,16.7)	0.03	11.5(8.6,16.2)	14.6 (10.9,19.4)	<0.01
NEU (%)	0.9 (0.8, 0.9)	0.9 (0.8, 0.9)	0.91	0.9 (0.8, 0.9)	0.9 (0.8, 0.9)	0.04
LYM (%)	0.1 (0.0, 0.1)	0.1 (0.0, 0.1)	0.88	0.1 (0.0, 0.1)	0.1 (0.0, 0.1)	0.06
REC (×10^12^/L)	3.5 (2.7, 4.1)	3.2 (2.4, 4.1)	0.10	2.8 (2.3, 3.7)	3.3 (2.5, 3.6)	0.27
HGB (g/L)	103.5 (78.0, 117.0)	91.0 (69.0, 111.0)	0.01	76.0 (64.0, 100.0)	91.0 (72.0, 108.5)	0.04
PLT (×10^9^/L)	176.0 (85.0, 259.0)	145.0 (71.0, 227.0)	0.08	142.0 (51.0, 206.0)	138.0 (79.0, 209.0)	0.50
CRP (mg/L)	157.2 (105.0,201.0)	150.0 (103.5, 201.0)	0.57	126.6 (67.3, 201.0)	140.5 (100.9, 198.6)	0.54
PCT (ng/mL)	1.7 (0.8, 7.1)	1.9 (0.7, 8.8)	0.61	2.8 (0.6, 8.3)	1.5 (0.6, 6.6)	0.46
Blood glucose			0.89			1.00
Normal	36 (28.6%)	32 (27.4%)		16 (37.2%)	25 (39.1%)	
Abnormal	90 (71.4%)	85 (72.6%)		27 (62.8%)	39 (60.9%)	
TBIL (μmol/L)	15.4(9.7,25.0)	18.2 (11.4, 33.2)	0.02	15.6(12.1,23.1)	15.4 (11.1, 25.1)	0.75
DBIL (μmol/L)	8.2(4.7,14.3)	9.0 (6.0, 18.8)	0.02	9.0 (5.1, 17.2)	8.6 (4.9, 13.9)	0.76
IBIL (μmol/L)	6.7(4.1,10.0)	7.7 (4.3, 12.0)	0.03	6.6 (4.1, 11.8)	6.8 (4.6, 12.0)	0.91
ALT (U/L)	28.5(15.0,52.0)	30.0 (18.0, 56.0)	0.69	33.0(17.0,75.0)	32.5 (19.1, 56.0)	0.47
AST (U/L)	42.0(28.0,71.0)	52.0 (33.0, 113.0)	0.02	53.7(34.0,92.0)	41.0 (27.0, 86.5)	0.26
BUN (mmol/L)	7.0(5.1, 11.0)	7.4 (4.9, 11.4)	0.63	7.4(5.5, 16.0)	6.8 (5.0, 11.9)	0.37
SCr (μmol/L)	90.0 (61.5, 138.0)	102.0 (71.0, 161.0)	0.01	103.0 (52.0, 147.0)	103.0 (68.0, 137.5)	0.94
K^+^ (mmol/L)	4.2 (3.7, 4.6)	4.2 (4.0, 4.9)	0.12	4.0 (3.7, 4.4)	4.0 (3.7, 4.6)	0.82
Na^+^ (mmol/L)	145.6 (139.5, 156.3)	144.1 (138.5, 153.2)	0.48	145.9 (138.4, 158.5)	144.8 (139.4, 153.8)	0.85
Ca^2+^ (mmol/L)	2.2 (2.1, 2.4)	2.2 (2.0, 2.4)	0.79	2.3 (2.1, 2.4)	2.2 (2.1, 2.4)	0.78
CPR			0.52			0.22
No	104 (82.5%)	92 (78.6%)		36 (83.7%)	59 (92.2%)	
Yes	22 (17.5%)	25 (21.4%)		7 (16.3%)	5 (7.8%)	

In the training cohort, the HA group showed significantly higher preoperative WBC counts (median 7.4 vs. 6.2 ×10^9^/L, P<0.01), elevated PCT levels (median 2.6 vs. 1.5 ng/mL, P<0.01), higher neutrophil (NEU) % (P = 0.04), lower HGB (P = 0.01), increased HPT (41.9% vs. 28.6%, P = 0.03), and more use of rATG induction therapy (7.7% vs. 1.6%, P = 0.02). FK506 trough concentrations were higher, though not statistically significant (P = 0.12).

In the validation cohort, only WBC count (P<0.01) and FK506 trough concentration (P = 0.01) remained significantly higher in the HA group, while other differences were not replicated. Donor characteristics were largely similar, except for higher bilirubin and creatinine levels in the training cohort’s HA group.

Overall, the two cohorts were comparable, with WBC count, FK506 concentration, and rATG use emerging as consistent differentiators, supporting their inclusion in predictive modeling.

### 3.3 Feature predictor selection

**Figure 2:**
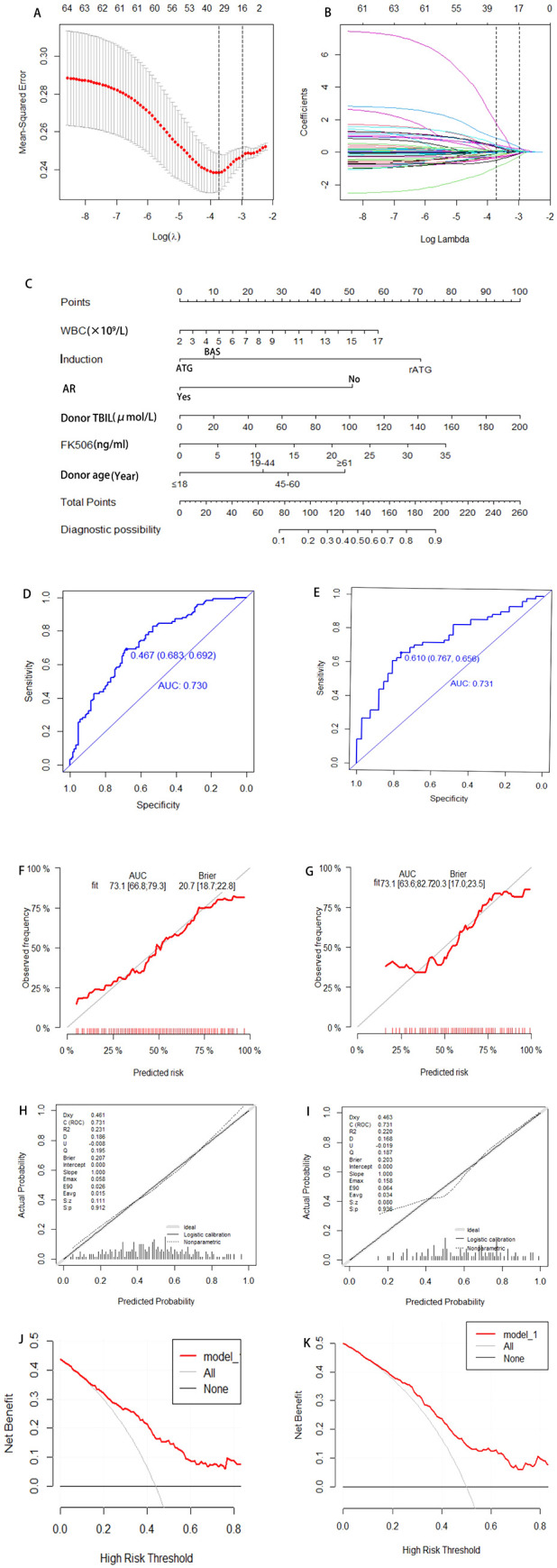
Development and validation a model for predicting risk factors of HA. Variable selection via LASSO regression with 10-fold cross-validation identifying optimal penalty parameter (λ) minimizing mean-squared error **(A)**. Corresponding coefficients of selected predictors at optimal λ **(B)**. A nomogram integrating independent risk factors including Preoperative WBC of recipients, induction, AR, donor TBIL, FK506, donor age, enabling individualized risk estimation **(C)**. ROC for training and validation cohort, with AUC of 0.730 and 0.731, respectively, indicating satisfactory discrimination **(D, E)**. Calibration plots comparing predicted and observed probabilities **(F, G)**; Hosmer-Lemeshow test P-values >0.05 indicate adequate model fit **(H, I)**. DCA demonstrating net benefit across a range of threshold probabilities compared to treat-all or treat-none strategies **(J, K)**. WBC, White Blood Cell; AR, Acute Rejection; AUC, Area Under the Curve; DCA, Decision Curve Analysis; ROC, Receiver Operating characteristic Curve; TBIL, Total Bilirubin; FK506, Tacrolimus.

This study employed univariate logistic regression, LASSO regularization algorithm (based on 10-fold cross-validation) combined with stepwise regression to screen potential predictive variables associated with HA after KT. As shown in **Table 3**, univariate logistic regression analysis identified 6 variables with significant differences between the normal and HA group. To further optimize variable selection, the LASSO regression was used to analyze all candidate variables. By adjusting the penalty coefficient λ (with the optimal λ value determined by minimizing the mean squared error), 14 candidate predictors were ultimately retained (**Figures 2A, B**). These variables screened by univariate Logistic regression and LASSO regression were incorporated into the multivariate Logistic regression model. The final model was selected based on the Akaike Information Criterion (AIC) combined with forward-backward stepwise regression, which balanced model fit and complexity. Six independent predictive factors were ultimately identified, including: recipient preoperative WBC count, induction, FK506 trough concentration, AR, donor age, and donor TBIL level. The results of the multivariate Logistic regression are detailed in **Table 4**.

**Table 3:** Univariate logistic regression analysis of the modeling cohort.

Variable	B	SE	Z	OR	95% CI	*P*-Value
Preoperative Recipient									
Gender	-0.456	0.280	2.662	0.634	0.366 - 1.096	0.103
Age (year)	0.383	0.220	3.030	1.466	0.953 - 2.256	0.082
Blood type									
A			3.294			0.348
B	0.496	0.574	0.749	1.643	0.534 - 5.058	0.387
O	0.981	0.577	2.886	2.667	0.860 - 8.268	0.089
AB	0.613	0.531	1.333	1.846	0.652 - 5.228	0.248
BMI (kg/m^2^)	-0.052	0.191	0.076	0.949	0.653 - 1.379	0.783
Hypertension	-0.287	0.367	0.614	0.750	0.366 - 1.539	0.433
HPT	0.589	0.272	4.682	1.801	1.057 - 3.070	0.030
Surgical history	-0.475	0.290	2.675	0.622	0.352 - 1.099	0.102
History of blood transfusion	-0.330	0.310	1.136	0.719	0.392 - 1.319	0.287
Smoking history	-0.293	0.334	0.774	0.746	0.388 - 1.434	0.379
Drinking history	-0.446	0.350	1.629	0.640	0.322 - 1.270	0.202
Dialysis modality									
No dialysis			5.329			0.149
HD	0.182	1.295	0.020	1.200	0.095 - 15.196	0.888
PD	-0.574	0.446	1.657	0.563	0.235 - 1.350	0.198
HD+PD	-1.322	0.599	4.875	0.267	0.082 - 0.862	0.027
Dialysis duration (month)	0.313	0.258	1.479	1.368	0.826 - 2.266	0.224
WBC (×10^9^/L)	0.150	0.053	7.828	1.161	1.046 - 1.290	0.005
NEU (%)	1.332	1.154	1.331	3.788	0.394 - 36.399	0.249
Blood calcium (mmol/L)	0.465	0.556	0.702	1.593	0.536 - 4.733	0.402
PCT (ng/ml)	0.005	0.014	0.114	1.005	0.977 - 1.033	0.735
CRP (mg/L)	-0.013	0.013	1.142	0.987	0.963 - 1.011	0.285
HLA antibodies	-0.063	0.534	0.014	0.939	0.329 - 2.675	0.906
Blood glucose	-0.244	0.336	0.529	0.783	0.406 - 1.513	0.467
Blood lipid	0.258	0.258	1.000	1.294	0.781 - 2.145	0.317
Perioperative Recipient									
Induction									
BAS			6.439			0.040
ATG	-1.516	0.797	3.617	0.220	0.046 - 1.047	0.057
rATG	-1.946	0.820	5.635	0.143	0.029 - 0.712	0.018
Maintenance regimen	0.806	0.626	1.655	2.239	0.656 - 7.641	0.198
Operation time (min)	0.106	0.257	0.168	1.111	0.671 - 1.841	0.682
AR	-1.090	0.592	3.384	0.336	0.105 - 1.074	0.066
DGF	0.320	0.398	0.647	1.377	0.632 - 3.002	0.421
Hormone pulse	0.223	0.370	0.363	1.250	0.605 - 2.584	0.547
FK506 trough concentration (ng/ml)	0.071	0.035	4.217	1.073	1.003 - 1.149	0.040
CRP (mg/L)	0.008	0.005	2.177	1.008	0.997 - 1.018	0.140
PCT (ng/mL)	0.015	0.008	3.639	1.015	1.000 - 1.031	0.056
WBC (×10^9^/L)	0.105	0.032	10.516	1.110	1.042 - 1.183	0.001
NEU (%)	5.235	2.966	3.116	187.801	0.561 - 62839.036	0.078
Hospital stay duration (day)	0.434	0.264	2.700	1.544	0.920 - 2.593	0.100
Donor									
Infectious marker	-0.684	0.454	2.274	0.505	0.207 - 1.228	0.132
Pathogen	-0.496	0.367	1.835	0.609	0.297 - 1.248	0.176
Gender	-0.042	0.320	0.017	0.959	0.512 - 1.795	0.896
Age (year)	0.394	0.173	5.194	1.482	1.057 - 2.079	0.023
BMI (kg/m^2^)	-0.043	0.216	0.040	0.958	0.628 - 1.462	0.842
WBC (×10^9^/L)	-0.041	0.019	4.472	0.960	0.925 - 0.997	0.034
NEU (%)	-0.090	0.888	0.010	0.914	0.160 - 5.210	0.920
LYM (%)	1.193	1.259	0.899	3.297	0.280 - 38.856	0.343
REC (×10^12^/L)	-0.198	0.122	2.604	0.821	0.646 - 1.043	0.107
HGB (g/L)	-0.010	0.004	5.457	0.990	0.982 - 0.998	0.019
PLT (×10^9^/L)	-0.002	0.001	2.101	0.998	0.996 - 1.001	0.147
CRP (mg/L)	-0.001	0.002	0.115	0.999	0.995 - 1.004	0.735
PCT (ng/mL)	0.005	0.005	0.758	1.005	0.994 - 1.015	0.384
TBIL (μmol/L)	0.021	0.009	6.146	1.022	1.004 - 1.039	0.013
DBIL (μmol/L)	0.022	0.011	4.073	1.022	1.001 - 1.043	0.044
IBIL (μmol/L)	0.058	0.021	7.757	1.060	1.017 - 1.104	0.005
ALT (U/L)	0.000	0.001	0.014	1.000	0.997 - 1.003	0.905
AST (U/L)	0.001	0.001	3.029	1.001	1.000 - 1.003	0.082
BUN (mmol/L)	0.038	0.025	2.429	1.039	0.990 - 1.019	0.119
SCr (μmol/L)	0.004	0.002	4.640	1.004	1.000 - 1.008	0.031
K^+^ (mmol/L)	0.033	0.112	0.086	1.033	0.830 - 1.286	0.770
Na^+^ (mmol/L)	-0.013	0.013	1.042	0.987	0.963 - 1.012	0.307
Ca^2+^ (mmol/L)	-0.154	0.563	0.074	0.858	0.284 - 2.587	0.785
CPR	0.250	0.325	0.592	1.285	0.679 - 2.431	0.442
Blood glucose	0.061	0.286	0.045	1.062	0.606 - 1.862	0.832
Cold ischemia time (hour)	0.051	0.257	0.040	1.053	0.636 - 1.741	0.842
ICU length of stay (day)	-0.181	0.257	0.495	0.835	0.504 - 1.381	0.482
OPO intervention time (hour)	0.469	0.260	3.256	1.599	0.960 - 2.661	0.071

**Table 4:** Multivariate logistic regression analysis of the modeling cohort.

Variable	B	SE	OR	95% CI	Z	*P* -value
Preoperative WBC of recipients (×10^9^/L)	0.142	0.057	1.15	1.03 - 1.29	2.506	0.012
Induction						
ATG (ref=BAS)	-0.379	0.315	0.68	0.37 - 1.27	-1.201	0.230
rATG (ref=BAS)	2.275	0.992	9.73	1.39 - 68.02	2.294	0.022
Donor TBIL (μmol/L)	0.018	0.009	1.02	1.00 - 1.04	2.021	0.043
FK506 trough concentration (ng/ml)	0.080	0.036	1.08	1.10 - 1.16	2.215	0.027
Donor age (year)	0.479	0.185	1.61	1.12 - 2.32	2.590	0.010
AR	-1.924	0.829	0.15	0.03 - 0.74	-2.320	0.020

### 3.4 Construction and validation of the nomogram

The final predictors were incorporated into the risk prediction model for post-KT HA (**Figure 2C**). In the training cohort, ROC analysis showed an AUC of 0.730, with a Youden index of 0.683. In the validation cohort, the model achieved an AUC of 0.731, with a Youden index of 0.767 (**Figures 2D, E**). To further assess model performance, 1000 Bootstrap resampling validation was conducted to confirm the model’s stability (**Figures 2F, G**). Calibration plots demonstrated excellent agreement between predicted and observed probability in both the training and validation cohorts, with midline deviations within clinically acceptable ranges. Brier scores and the coefficient of determination (R²) were used to quantify the model’s calibration performance: the training cohort achieved a Brier score of 0.207 (P = 0.912) with R²=0.231, while the validation cohort showed a Brier score of 0.203 (P = 0.936) with R²=0.220, indicating the satisfactory calibration and discrimination (**Figures 2H, I**). In addition, Additionally, DCA results showed that the model provided clinical benefit across a wide range of threshold probabilities, supporting its potential application in clinical practice. For both the training and validation cohorts, the model demonstrated robust discrimination and calibration, suggesting it can reliably identify patients at risk for post-transplant HA and support individualized risk assessment (**Figures 2J, K**).

## 4 Discussion

This study developed and internally validated a multifactorial nomogram to predict the risk of HA following KT. By integrating recipient inflammatory status, immunosuppressive protocols, and donor factors, the model provided crucial knowledge in perioperative management. Practical application of this model enables early identification of high-risk individuals, facilitating targeted monitoring and intervention, which may help reduce postoperative complications and optimize graft outcomes.

Recipient preoperative WBC count was identified as a significant independent predictor of post-transplant HA, underscoring the central role of systemic inflammation in early metabolic disturbances post-KT. Elevated preoperative WBC reflected inflammation state, which was associated with adverse outcomes in KT, including graft dysfunction and increased susceptibility to complications (17–19). Previous studies have linked inflammatory markers such as CRP and NEU counts to post-transplant metabolic disturbances. (20, 21). Inflammatory response may damage pancreatic tissue through the inflammatory cascade and induction of apoptosis and necrosis, which supports the role of inflammation in pancreatic enzyme elevation (22, 23). By incorporating preoperative WBC into our model, we provided quantifiable evidence that inflammation was a key factor in HA risk, emphasizing the need for close monitoring of inflammatory status in the early post-transplant period. This finding aligned with and extended existing knowledge by directly associating WBC with HA risk (24).

Immunosuppressive regimen, particularly rATG induction and higher FK506 trough concentration, also contributed to increased HA risks. Potent immunosuppression can heighten susceptibility to metabolic complications, either by immune-mediated mechanisms or direct pancreatic toxicity. Prior literature has documented FK506-associated HA and pancreatitis in KTR (25–27). This phenomenon may be linked to FK506-induced activation of the mitochondrial-dependent apoptosis pathway, which leads to the disruption of mitochondrial membrane integrity in pancreatic exocrine cells and the abnormal accumulation of reactive oxygen species. Consequently, this process results in the upregulation of pro-apoptotic proteins and apoptotic executioner proteins, ultimately precipitating exocrine cell dysfunction and, in some cases, cell death. (28). The reduction in the incidence of acute rejection (AR) was probably associated with the elevated trough concentrations of FK506 in HA group, which, in turn, align with its well-established immunosuppressive properties. Adequate FK506 exposure effectively suppresses alloreactive immune responses, thereby attenuating the recipient’s immune-mediated AR-related injury to the transplanted graft. Unlike earlier models that focused primarily on recipient clinical variables, our study uniquely integrated detailed immunosuppressive parameters, enhancing the clinical relevance and applicability of the predictive model. These results underscored the importance of optimizing immunosuppressive dosing to balance rejection prevention with metabolic safety.

Beyond recipient and treatment-related factors, donor characteristics further contributed to HA risk. Donor age and TBIL emerged as significant predictors in our model, highlighting the influence of donor organ quality on recipient outcomes. Advanced donor age has been consistently associated with reduced graft function and a higher risk of complications (29, 30). Elevated donor TBIL, reflecting hepatic and systemic health, has been linked to oxidative stress and immune modulation, which might affect graft viability and recipient metabolism (31–33). Our findings corroborated these associations and extended prior research by integrating donor biochemical markers into HA risk assessment. This comprehensive approach improved prediction accuracy and supported personalized transplant management strategies. Together, these findings demonstrated the multifactorial nature of post-transplant HA, involving recipient inflammation, immunosuppressive therapy, and donor factors.

Subsequently, our findings suggested that HA may function more as a marker of perioperative metabolic imbalance. Nevertheless, the clinical significance of isolated HA in the absence of other complications remains uncertain, largely due to the lack of standardized diagnostic criteria and the multifactorial nature of its underlying mechanisms. Future research, particularly large-scale, multicenter studies with long-term follow-up, is required to determine the extent to which HA influences long-term outcomes, including graft survival and overall patient survival.

This study has several limitations. First, the single-center retrospective design may introduce selection bias and limit the generalizability. Future multicenter, prospective studies are necessary to validate the model in diverse populations. Second, some important factors, such as nutritional status, genetic predispositions, and dynamic changes in clinical parameters, were unavailable and could improve prediction if included. Third, although internal validation demonstrated robust model performance, external validation using independent datasets remains essential before clinical use. Addressing these issues is key to applying the model in practice.

Despite its limitations, this study introduces an innovative and comprehensive predictive model for assessing the risk of HA following KTR. By integrating recipient inflammatory biomarkers, immunosuppressive therapy details, and donor biochemical indicators, we have developed a risk stratification model that facilitates timely and personalized patient assessments. This model enables the early identification of high-risk individuals, thereby providing robust support for tailored patient management. Clinicians can optimize treatment strategies through enhanced monitoring, adjustments to immunosuppressive regimens, ensuring adequate hydration, and closely monitoring amylase levels, all of which contribute to effective complication prevention. While further multicenter prospective validation is necessary, the development of this tool represents a significant advancement in precision perioperative care for KTR. Future research should focus on external validation and the incorporation of dynamic biomarker monitoring alongside multi-omics data to further refine the risk prediction model.

## 5 Conclusion

This study provides a novel and comprehensive prediction model for post-transplant HA in KTR. This prediction model could help identify high-risk populations for post-transplant HA and provides support for personalized patient management. Our study provides a foundation for improving transplant outcomes through individualized care.

## Data Availability

The original contributions presented in the study are included in the article/supplementary material. Further inquiries can be directed to the corresponding author.
